# Osteosarcoma and Ewing Sarcoma of Bone: An Italian Mono-Institutional Epidemiological Study

**DOI:** 10.3390/diagnostics15030328

**Published:** 2025-01-30

**Authors:** Cristina Ferrari, Giovanna Magagnoli, Roberta Laranga, Giuseppe Bianchi, Elisa Carretta, Marilena Cesari, Katia Scotlandi, Nicola Baldini, Davide Maria Donati, Marco Gambarotti

**Affiliations:** 1 Laboratory of Experimental Oncology, IRCCS Istituto Ortopedico Rizzoli, 40136 Bologna, Italy; cristina.ferrari@ior.it (C.F.); katia.scotlandi@ior.it (K.S.); 2 Department of Pathology, IRCCS Istituto Ortopedico Rizzoli, 40136 Bologna, Italy; giovanna.magagnoli@ior.it (G.M.); marco.gambarotti@ior.it (M.G.); 3 Unit of 3rd Orthopaedic and Traumatologic Clinic Prevalently Oncologic, IRCCS Istituto Ortopedico Rizzoli, 40136 Bologna, Italy; giuseppe.bianchi@ior.it (G.B.); davidemaria.donati@ior.it (D.M.D.); 4 Department of Programming and Monitoring, IRCCS Istituto Ortopedico Rizzoli, 40136 Bologna, Italy; elisa.carretta@ior.it; 5 Osteoncology, Soft Tissue and Bone Sarcomas, Innovative Therapy Unit, IRCCS Istituto Ortopedico Rizzoli, 40136 Bologna, Italy; marilena.cesari@ior.it; 6 Orthopedic Pathophysiology and Regenerative Medicine Unit, IRCCS Istituto Ortopedico Rizzoli, 40136 Bologna, Italy; nicola.baldini@ior.it; 7 Department of Biomedical and Neuromotor Sciences (DIBINEM), University of Bologna, 40123 Bologna, Italy

**Keywords:** bone osteosarcoma, ewing sarcoma of bone, geographic pattern

## Abstract

**Background/Objectives**: Musculoskeletal neoplasms are rare and challenging diseases. Their geographic pattern varies worldwide, and no studies analyze their distribution in Italy. The aim of this study was to investigate a possible association between clinical variables to a period of diagnosis and geographic origin in Italy. Moreover, we wanted to describe the survival rate of bone osteosarcoma (OS) and Ewing sarcoma (EwS) from the Rizzoli Orthopaedic Institute (IOR) experience. **Methods**: We retrospectively reviewed 3098 diagnoses of high-grade bone OS and EwS made at the IOR in the past 40 years (1982–2021). Incidence, measures of associations, and survival rates have been analyzed. **Results**: The time of diagnosis and geographic origin were associated either with each other or with age and stage of tumor. Overall, the 10-year survival rate was 54% (95% CI 52–56) and 53% (95% CI 50–56) for bone OS and EwS, respectively. Multivariate analyses showed that adverse factors at diagnosis are age, location, stage, and time of diagnosis, in both cohorts. **Conclusions**: We confirmed known prognostic factors, and owing to the large cohort, we highlight their importance in clinical practice. No differences were observed in patient survival associated with different areas of Italy, although geographic origin was associated with most clinical variables analyzed, suggesting a further factor to investigate. Given the above-mentioned results, a Sarcoma Specialist Network with a recognized expertise is determinably in charge of the management of sarcomas.

## 1. Introduction

Primary malignant bone tumors are rare neoplasms, and they account for 3 to 5% of cancers in children and adolescents [1,2,3,4]. Bone osteosarcoma (bone OS) is the most frequent primary bone sarcoma (incidence: 0.3 per 100,000 per year). Its incidence is higher in adolescents (0.8–1.1 per 100,000 per year at age 15–19 years) with a male to female ratio of 1.4:1 [5,6]. In younger patients, bone OS arises mainly in correspondence with extremities, while the number of axial tumors increases with age [7]. Ewing sarcoma (EwS) is the second most common bone sarcoma in children, adolescents, and young adults and belongs to the group of ‘small round blue cell tumors’, showing aggressive behavior [8,9,10]. The median age at diagnosis is 15 years with male predominance (1.5:1) [7].

In Italy, bone sarcomas make up 9% of all sarcomas: bone OS and EwS of bone have their highest incidence at ages 10–19 [11]. The incidence of primary bone sarcoma in Italy averages around 1 case per 100,000 people each year, corresponding to 350 new cases per year; there are about 120 new diagnoses of bone OS/year, and 65 new cases/year of Ewing Sarcoma (AIRC Foundation for Cancer Research).

Prognosis has improved markedly over the last decades, thanks to more specific diagnosis and treatments; however, these diseases remain challenging [12,13]. The Rizzoli Orthopedic Institute (IOR) is the leading reference center in the diagnosis and treatment of sarcomas in Italy. In the last 40 years, we registered 40% of Italian bone OS cases and 46% of Ewing sarcoma Italian cases at the IOR.

The purpose of this work is to describe the characteristics and clinical outcomes of patients admitted for treatment to the IOR from the early 1980s to the present. Moreover, considering that in the 80s–90s, the IOR was the first musculoskeletal oncologic center in Italy and later one of the leading local centers; we also described clinical variables and outcomes according to the geographical origin of patients.

## 2. Materials and Methods

### 2.1. Patients

This is a retrospective cohort study including patients diagnosed and treated for high grade bone OS and EwS at the Rizzoli Orthopedic Institute (IOR), from 1982 to 2021. The study was approved by the Local Ethical Committee (CE-AVEC; Prot.No.641/2023/Oss/IOR) on 3 November 2023.

The clinical data were collected from the database of the Rizzoli Orthopaedic Institute (recognized by the Emilia Romagna region by Regional Council No. 862 of 7/3/95) [14].

This Database collects information of sarcoma patients treated at the IOR since January 1982, with the aim of performing clinical research. The data collected are prevalently clinical-pathologic and include medical history, diagnosis, tumor site, surgery, treatment, and follow-up. Histological diagnosis was performed according to WHO current classification of soft tissue and bone tumors [15], by experienced pathologists of the IOR. The diagnosis previously made in other hospitals was further confirmed at the IOR, and patients were not formerly treated elsewhere. Follow-up data and patient status were updated on 31 December 2023, through a digital archive.

**Figure 1:**
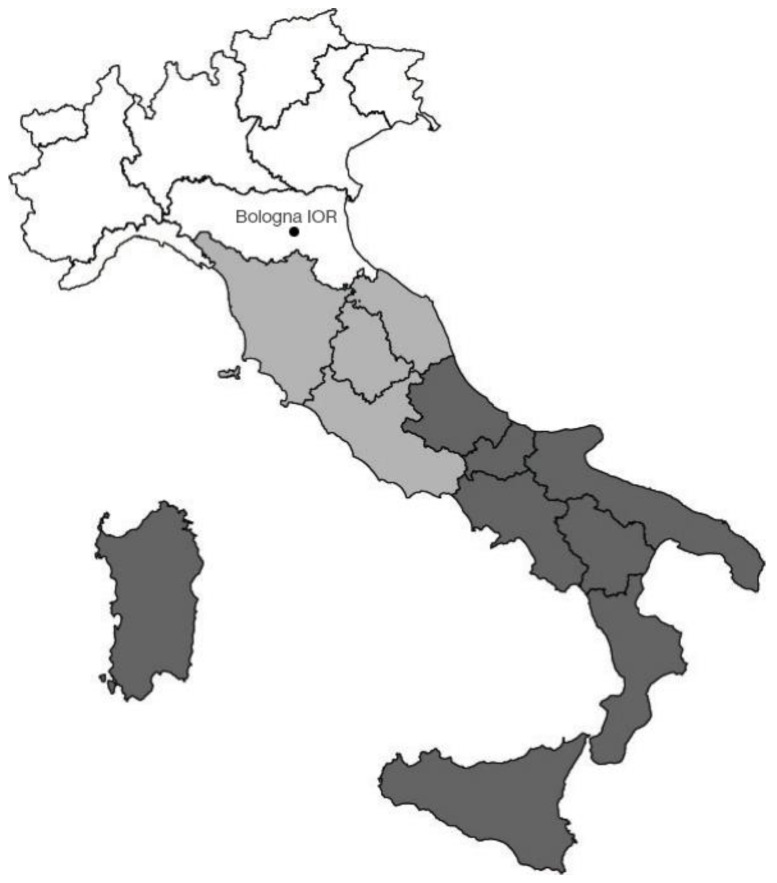
The three geographical zones of Italy: north (white), center (light gray), and south and islands (gray) www.istat.it/statistiche-per-temi/ambiente-e-territorio/ (accessed on 16 January 2025).

Clinical data included patient characteristics (age, gender), tumor characteristics (site, stage), patient demographics (year of diagnosis, area of origin), and clinical outcome (status at last follow-up). We followed the age stratification employed by our clinicians, dividing patients into three groups: pediatric (ages 0–14), young adults (ages 15–29), and adults (ages 30 and above). The 20 Italian regions were grouped into three distinct geographical zones: north, center, and south and islands (Figure 1).

We have adopted the subdivision of Italy in 3 macro areas (north, center, and south and islands) according to the ISTAT (Italian National Institute of Statistics) [16]. This classification is founded on economic, climatic, and geographic attributes and represents the most prevalent approach to delineate the Italian territory in various types of analyses, including epidemiological studies [17,18]. Istituto Ortopedico Rizzoli is situated in Bologna, within the Emilia Romagna region, which is in central-northern Italy. Its central location renders it a key destination that is readily accessible from all regions of Italy.

### 2.2. Statistics

A total of 1893 patients with bone OS and 1205 patients with EwS treated at the IOR (1982–2021) were analyzed by age (<15, 15–29, >30 years), gender (M, F), stage (localized or metastatic disease at diagnosis), tumor site (lower extremity, upper extremity, or axial), time of diagnosis (1982–1991, 1992–2001, 2002–2011, 2012–2021), and geographic origin (northern Italy, central Italy, southern Italy and islands, and foreign countries). Measures of association were estimated by the chi-square test or Fisher’s exact test for association between categorical variables and by the Cochran–Mantel–Haenszel test (CMH) for the analysis of stratified or matched categorical data. Patient characteristics were reported as mean along with range and as percentages for continuous and categorical variables, respectively. All variables were analyzed for their impact on overall survival (OS). Patients with a missing follow-up (bone OS = 9 and EwS = 5) or those who were residents abroad (bone OS = 149 and EwS = 87) were excluded from the survival analysis. OS was calculated from diagnosis to death for any cause or to the date of the last follow-up examination. OS estimates by univariate analysis were calculated according to the Kaplan–Meier method. A log-rank test was used for the calculation and comparison of survival curves. Hazard ratios and confidence intervals (95%) were calculated using the Cox hazards model. The statistical analysis was performed with Stata software version 17.0.

## 3. Results

### 3.1. Patients Characteristics

In the reviewed 40-year period, we identified 3098 patients with a diagnosis of bone OS and EwS. In detail, the diagnosis was bone OS for 1893 patients and EwS for 1205. A descriptive analysis is summarized in Table 1.

The average age of patients with bone OS was 21 years (range 2–84 years) and 19 years (range 0–73 years) for EwS. According to gender, we found 1125 (60%) males and 768 (40%) females for bone OS; we found 789 (65%) males and 416 (35%) females for EwS.

Among bone OS cases, 78% (1478) arise in the lower extremity, 12% (231) in the upper extremity, and 10% in the axial compartment. Regarding EwS patients, tumor onset is mostly in correspondence with the lower extremity (542; 45%) and axial sites (523; 43%), followed by the upper extremity (140; 12%). Of the 1893 patients with bone OS, 19% of tumors were metastatic at diagnosis. Considering the 1205 Ewing patients, 24% presented with metastases at diagnosis.

Each group of patients was stratified by geographic origin. Across the bone OS dataset, 728 (38%) came from northern Italy, 296 (16%) from central Italy, 720 (38%) from southern Italy and islands, and 149 (8%) from abroad. Amongst EwS patients, 472 (40%) came from northern Italy, 160 (13%) came from central Italy, 486 (40%) came from southern Italy and islands, and 87 (7%) from abroad.

Patients were divided into decades according to the year of diagnosis: 1982–1991, decade 1; 1992–2001, decade 2; 2002–2011, decade 3; and 2012–2021, decade 4. Clinical variables stratified by decade of diagnosis are reported in Table 2 for bone OS and Table 3 for EwS.

**Figure 2:**
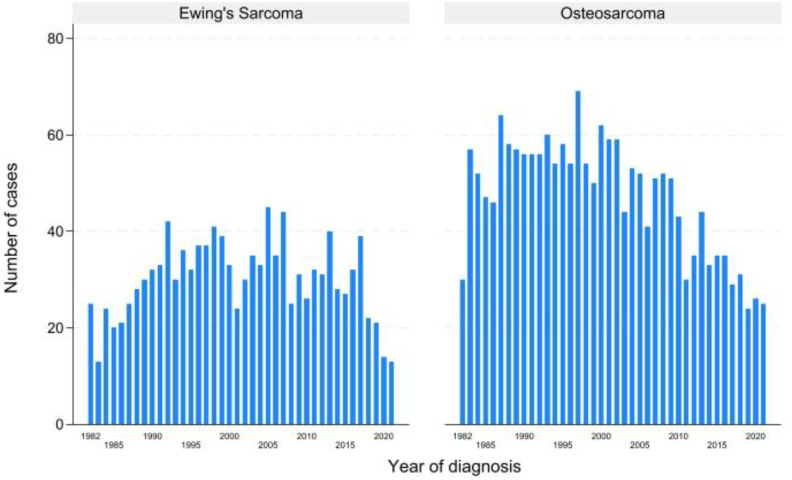
Distribution of cases per year of diagnosis.

The incidence of the two pathologies per year at the IOR is shown in Figure 2.

### 3.2. Demographics

Characteristics of the two cohorts of patients were analyzed considering their geographic origin. For bone OS, all variables, but gender, resulted as statistically significant when associated with geographic origin (Table 4).

Concerning the group of patients affected by EwS, there were no significant differences between origin concerning gender and stage; age resulted significantly associated with geographic origin (*p* = 0.0436) (Table 5).

### 3.3. Survival Analysis

#### 3.3.1. Bone OS

Of 1735 patients affected by bone OS, the cumulative percentage of overall survival (OS) was 59% (95% CI 57–62) and 54% (95% CI 52–56) at the 5- and 10-year follow-up, respectively. Survival analyses are reported in Table 6.

**Figure 3:**
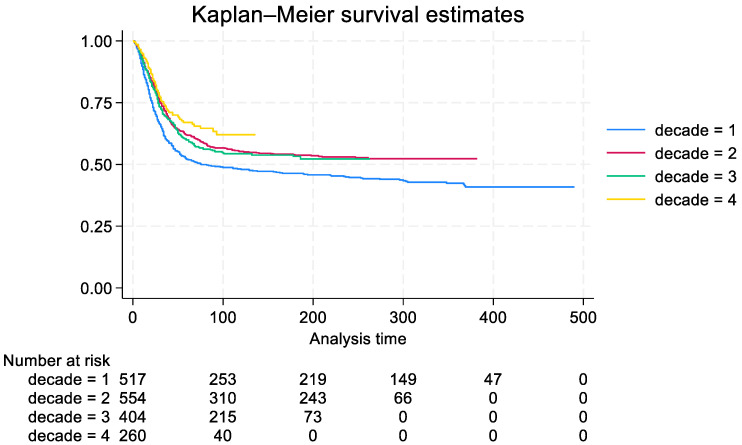
Overall Survival estimate in bone OS for patients stratified by decade of diagnosis (years). Decade 1, 1982–1991; decade 2, 1992–2001; decade 3, 2002–2011; decade 4, 2012–2021.

Age, gender, tumor site, stage, and decade of diagnosis are independent risk factors associated with OS (Table 4). Patients over 30 years old have a higher risk of death compared to younger ones (HR 1.59 *p* < 0.001). Male patients have a lower survival compared to females (HR 1.22 *p* = 0.006). Axial tumors have a worse prognosis than tumors in the lower extremity (HR 2.56 *p* < 0.001). The metastatic stage is associated with a higher risk of death (HR 4.44 *p* < 0.001). Furthermore, diagnoses in the first decade (1982–1991) are correlated to a worse prognosis compared to more recent diagnoses (2 vs. 1 HR 0.61 *p* < 0.001; 3 vs. 1 HR 0.62 *p* < 0.001; 4 vs. 1 HR 0.33 *p* < 0.001) (shown in Figure 3).

A significant difference was found comparing decade 2 to decade 4 (HR 1.8 *p* < 0.001 95% CI 1.4–2.3). Finally, geographic origin did not correlate with survival (*p* = 0.4329). Metastases at onset represent a risk factor for all patients with bone OS; by stratifying them by decade of diagnosis we underlined how this negative prognosis has improved over the years, going from a 5-year OS of 10% (95% CI 0.04–0.18) in the first decade to 40% in the fourth decade (95% CI 0.27–0.52). Similar results were found for patients with localized bone OS (Table 7).

#### 3.3.2. Ewing Sarcoma

Considering a total of 1113 patients affected by EwS, the cumulative percentage of survival was 59% (95% CI 56–61) and 53% (95% CI 50–56) at 5- and 10-year follow-up, respectively. Survival estimate analyses are reported in Table 8.

Age, tumor site, stage and time of diagnosis resulted as independent risk factors when associated with survival. Precisely, age between 15 and 29 years or over 30 affected survival (HR 1.45 *p* < 0.001 and HR 2.01 *p* < 0.001, respectively). Tumor onset in the lower extremities resulted in being less risky than axial development (HR 0.68 *p* < 0.001). The metastatic stage was associated with poorer outcome (HR 3.78 *p* < 0.001), a result confirmed in all decades (Table 9).

**Figure 4:**
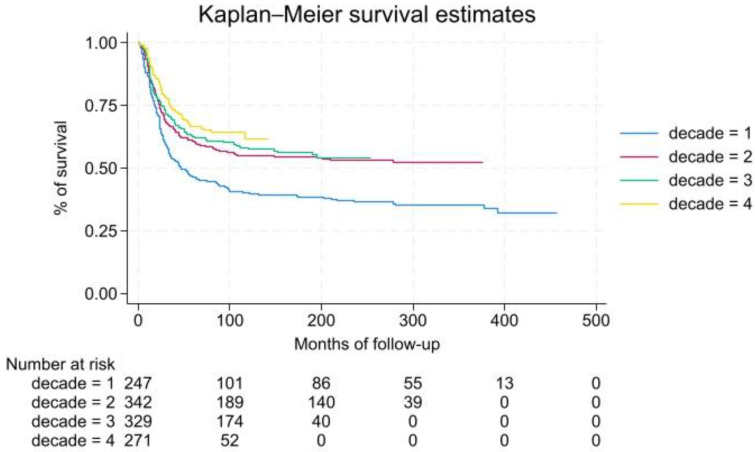
OS estimate for EwS patients stratified by decade of diagnosis. Decade 1, 1982–1991; decade 2, 1992–2001; decade 3, 2002–2011; decade 4, 2012–2021.

Diagnoses made in the first decade have a worse prognosis compared to more recent diagnoses (2 vs. 1 HR 0.52 *p* < 0.001; 3 vs. 1 HR 0.43 *p* < 0.001; 4 vs. 1 HR:0.30 *p* < 0.001) (Figure 4).

In terms of survival, a significant difference was found comparing decade 2 to decade 4 (HR 1.69 *p* < 0.0001 95% CI 1.28–2.23). Geographic origin did not correlate with survival. Finally, comparing patients with bone OS and EwS there were no significant differences in terms of survival either considering the whole cohort of patients (*p* = 0.3720) or limiting the analysis to patients with localized disease (*p* = 0.83).

## 4. Discussion

This study investigates clinical and demographic data of patients affected by bone OS and EwS in Italy, treated at the IOR in the last 40 years (1982–2021). Previous studies have explored the relationship between socioeconomic factors and risk of presenting with known bone tumor-related prognostic factors [19]. There are available international data for bone OS and EwS assessing the association between rurality and oncological outcomes while controlling for crucial socioeconomic factors [20,21]. However, no studies have completed a combined analysis to determine if there is an association between geographic provenience and clinical characteristics/outcome of bone OS or EwS in Italy. For this purpose, we collected data from the IOR in the last 40 years (1982–2021), to improve the availability of information on these rare diseases.

The overall incidence of patients diagnosed and treated at the IOR increased between 1982 and 2001 for both types of sarcoma, confirming Rizzoli’s primacy in Italy for musculoskeletal pathology treatment in the 1980s and 1990s, immediately after the introduction of neoadjuvant chemotherapy [22,23]. After 2000, however, there was a decline in attendance at our Institute, and this can certainly be attributed to the emergence of others sarcoma treatment centers in Italy.

Decades of diagnosis and clinical variables underline an increased trend in metastatic diagnoses, in the proportion of people diagnosed after the age of 30, and in patients coming from abroad. We believe that this is due to the necessity of metastatic patients to be addressed at specific sarcoma treatment center, such as the IOR (second-level treatment center) [23,24]. Instead, the lower number of pediatric patients counted in the years could be due to the spread of others treatment centers in Italy.

After 2000, there was a decline in patients treated at the IOR regardless of the region of origin. An opposite trend is observed for foreign patients, probably related to increased travel feasibility and as well to the advancement of internet communication that has facilitated access to clinical information. Geographic origin was also associated with age in both cohorts of patients, with a higher prevalence of younger patients, possibly due to the necessity of more specific treatments provided only in sarcoma reference centers [24]. This trend confirms the pivotal contribution of IOR till 2000 in the management of sarcomas in Italy across all ages.

Among bone OS, the analysis indicates a significant association between regions of origin and tumor stage. We noted that metastatic patients coming from the north and from the center of Italy were lower compared to the localized ones, perhaps thanks to quicker diagnosis, considering their proximity to the IOR. However, these geographical disparities can also be explained by differences in socioeconomic and medical status. To our opinion, changing incidence could result from numerous factors, including completeness of registration, population composition, and exposure to risk factors. The association between region of provenance and other clinical variables suggests a possible role of this variable and highlights a further factor to consider. Monitoring these trends is important for planning healthcare delivery and for prevention screening and monitoring.

Survival analysis showed that the 5-year and 10-year OS for bone OS patients was 59% (95% CI 57–62) and 54% (95% CI 52–56), respectively. For patients with EwS, the 5-year and 10-year OS corresponded to 59% (95% CI 56–61) and 53% (95% CI 50–56), respectively. These results were comparable to previously reported data [25,26].

In agreement with other studies [27,28,29], our findings showed that age is a significant risk factor associated with survival. Patients younger than 14 years have a more favorable prognosis compared to older ones, either due to a better response to chemotherapy treatment, despite the higher dosage usually administrated to them, or to the absence of comorbidities.

We observed that bone OS and EwS occur more often in males compared to females, even if gender resulted in being a risk factor associated with survival in bone OS but not in EwS. Other analyses have also reported a similar trend either in bone OS [26] or in EwS [30]. However, it should be considered that gender disparities in overall tumor incidence and life expectancy are significantly influenced by socioeconomic and cultural factors, exhibiting varied distribution across different global regions. Both statistical analyses and historical evidence consistently indicate that social inequalities between genders play a crucial role in shaping these patterns [31].

In general, 80% to 90% of bone OS cases occur in long tubular bones, and the axial skeleton is rarely affected [32]. EwS of the bone mostly affects the diaphysis and has a predilection for long and axial bones [33]. Our analysis shows that for both types of sarcomas, the site is a significant risk factor associated with survival. Tumors localized into the extremities have a better course than the axial ones. This, according to previous studies [26,32], is certainly related to a more effective surgical approach, often with adequate margins.

Survival rates have evolved over the past four decades (*p* < 0.001), a conclusion that is further corroborated by Italian cancer epidemiological data [11].

The factors contributing to this outcome are numerous and, in our view, interrelated, to the evolution of a multidisciplinary approach in oncology. This method began to take shape in the mid-1980s, when it was demonstrated that incorporating chemotherapy alongside radiotherapy and/or surgery significantly enhanced patient survival [34]. In recent decades, there has been a significant transformation in the detection and treatment of cancer, facilitated by advancements in imaging technology (MRI/CT scan) and molecular biological techniques [12,35]. Advances in technology and biological understanding highlight that interdisciplinary collaboration is key.

Regarding the medical management of osteosarcoma and Ewing’s sarcoma, there has been little significant change in chemotherapy from the early 1980s to the present. While some treatment protocols have undergone minor modifications or implementations, these adjustments have had minimal effect on patient prognosis [36,37]. A comparable observation can be made regarding surgical techniques. Nevertheless, during this period, limb-sparing surgical strategies began to be favored over more invasive methods, such as amputation, particularly when combined with neo-adjuvant chemotherapy [38,39].

In summary, the synergic collaboration of various professionals yields significant advantages in the care of cancer patients, leading to enhanced quality of life and, ultimately, better treatment outcomes and survival rates.

In our case series, metastatic sarcomas at diagnosis were 19% in bone OS and 24% in EwS, with a clear prevalence of lung metastases in both cases (75% and 51%). As expected, metastases at onset are a poor prognostic sign for all patients [40,41].

Regardless of the tumor stage, both bone OS and EwS showed a change in survival over the decades of diagnosis. Despite this, the prognosis is still not good for metastatic patients. This improvement in prognosis may be explained with the introduction of increasingly effective therapies combined with more accurate and precise diagnoses including genetic and molecular techniques [13]. However, caution should also be taken in comparing historical data, since more refined imaging techniques have led to a stage shift over the last decades.

Comparing the survival curves between the second and fourth decades of diagnoses, since the 1990s, Rizzoli was almost the only referral center for musculoskeletal tumors in Italy. We highlighted a significant difference. Nevertheless, the reduced follow-up for patients in the fourth decade should be considered. Finally, geographic provenience was not an independent risk factors for mortality. This result is in contrast with other published data, where the distance to the nearest comprehensive center affects survival outcomes [20,21].

This retrospective analysis also aimed to investigate patient flow at the IOR from different geographic areas of Italy, presuming a higher turnover from the bordering regions. However, no difference was observed; the number of patients coming from the north and south of Italy was very similar, while the number of patients coming from the center was lower, to a lower population and a smaller geographical area.

The retrospective design of the study and the long period covered are the main weaknesses of this study. Due to the nature of a retrospective cohort, the accuracy of historical data can give potential bias in the data collection. Furthermore, certain variables such as socioeconomic ones are lacking, and this can be important to consider when evaluating differences between region of provenience and accessibility to healthcare. However, to our knowledge, this review is the largest bone OS and EwS study performed to date in Italy. The large number and broad eligibility criteria of patients, which include patients with axial or metastatic disease, extend the relevance of our findings compared to most other musculoskeletal tumor analyses.

Finally, these relevant numbers demonstrate an almost half-century experience of the Rizzoli Institute in the treatment of bone sarcomas despite the rareness of these pathologies; moreover, this experience includes extensive expertise in the treatment of pediatric patients. Therefore, in our opinion the management of sarcomas should be performed by a Sarcoma Specialist Network, with strong knowledge, as demonstrated here.

## 5. Conclusions

The findings of this study acknowledge and establish once again the fundamental role in the treatment of rare diseases and the role of specialized centers with multidisciplinary expertise. We confirmed known prognostic factors, and thanks to the considerable number of patients included, we underlined their role in clinical practice. Despite the absence of correlation between geographic origin and survival, we validated that it is associated with relevant clinical variables. Future studies should aim to determine its role in these diseases.

## Figures and Tables

**Table 1:** Descriptive analysis of Bone OS and EwS patients.

		Bone OS (N 1893)	Ewing Sarcoma (N 1205)
Variable	Level	N (%)	N (%)
Age at diagnosis	0 ≤ X ≤ 14	729 (39)	470 (39)
	15 ≤ X ≤29	858 (45)	574 (48)
	X ≥ 30	306 (16)	161 (13)
Gender	Female	768 (40)	416 (35)
	Male	1125 (60)	789 (65)
Tumor site	Lower	1478 (78)	542 (45)
	Upper	231 (12)	140 (12)
	Axial	184 (10)	523 (43)
Stage	Localized	1532 (81)	916 (76)
	Metastatic	361 (19)	289 (24)
Geographic origin	North	728 (38)	472 (40)
	Center	296 (16)	160 (13)
	South and islands	720 (38)	486 (40)
	Abroad	149 (8)	87 (7)
Decade	1982–1991	523 (28)	248 (21)
	1992–2001	574 (30)	345 (29)
	2002–2011	477 (25)	336 (28)
	2012–2021	319 (17)	276 (23)

**Table 2:** Descriptive analysis of clinical variables stratified by decade of diagnosis for bone OS.

		1982–1991	1992–2001	2002–2011	2012–2021	*p*-Value
Variable	Level	N (%)	N (%)	N (%)	N (%)	
Age	0 ≤ x ≤ 14	233 (45)	194 (34)	180 (38)	122 (38)	<0.001
	15 ≤ x ≤ 29	245 (47)	280 (49)	207 (43)	126 (39)	
	x ≥ 30	45 (9)	100 (17)	90 (19)	71 (22)	
Gender	Female	230 (44)	230 (40)	196 (41)	112 (35)	0.087
	Male	293 (56)	334 (58)	281 (59)	207 (65)	
Stage	Localized	458 (88)	466 (81)	374 (78)	234 (73)	<0.001
	Metastatic	65 (12)	108 (19)	103 (22)	85 (27)	
Geographic origin	North	212 (41)	209 (36)	174 (36)	133 (42)	<0.001
	Center	94 (18)	97 (17)	71 (15)	34 (11)	
	South and islands	211 (40)	250 (44)	163 (34)	96 (30)	
	Abroad	6 (1)	18 (3)	69 (14)	56 (18)	

**Table 3:** Descriptive analysis of clinical variables stratified by decade of diagnosis for EwS.

		1982–1991	1992–2001	2002–2011	2012–2021	*p*-Value
Variable	Level	N (%)	N (%)	N (%)	N (%)	
Age	0 ≤ x ≤ 14	93 (38)	134 (39)	137 (41)	106 (38)	0.009
	15 ≤ x ≤ 29	137 (55)	168 (49)	142 (42)	127 (46)	
	x ≥ 30	18 (7)	43 (12)	57 (17)	43 (16)	
Gender	Female	86 (35)	131 (38)	115 (34)	84 (30)	0.276
	Male	162 (65)	214 (62)	221 (66)	192 (70)	
Stage	Localized	209 (84)	278 (81)	246 (73)	183 (66)	<0.001
	Metastatic	39 (16)	67 (19)	90 (27)	93 (34)	
Geographic origin	North	102 (41)	143 (41)	121 (36)	106 (38)	<0.001
	Center	36 (15)	49 (14)	47 (14)	28 (10)	
	South and islands	108 (44)	143 (41)	133 (40)	102 (37)	
	Abroad	2 (1)	10 (3)	35 (10)	40 (14)	

**Table 4:** Population characteristics by geographic origin in the bone OS patient cohort.

		Geographic Origin				*p*-Value
		North N (%)	Center N (%)	South/Islands N (%)	Abroad N (%)	
Gender	F	296 (40.66)	127 (42.91)	238 (39.31)	62 (41.61)	0.7491
	M	432 (59.34)	169 (57.09)	437 (60.69)	87 (58.39)	
Age	0–14	242 (33.24)	132 (44.59)	291 (40.42)	64 (42.95)	<0.001
	15–29	329 (45.19)	120 (40.54)	333 (46.25)	76 (51.01)	
	30+	157 (21.57)	44 (14.86)	96 (13.33)	9 (6.0)	
Stage	Localized	604 (82.97)	246 (83.11)	572 (79.44)	110 (73.83)	0.0217
	Metastatic	124 (17.03)	50 (16.89)	148 (20.56)	39 (26.17)	

**Table 5:** Population characteristics by geographic origin in the EwS patient cohort.

		Geographic Origin				*p*-Value
		North N (%)	Center N (%)	South/Islands N (%)	Abroad N (%)	
Gender	F	161 (34.11)	56 (35)	168 (34.57)	31 (35.63)	0.9919
	M	311 (65.89)	104 (65)	318 (65.43)	56 (64.37)	
Age	0–14	162 (34.32)	65 (40.62)	206 (42.39)	37 (43.53)	0.0436
	15–29	232 (49.15)	73 (45.62)	225 (46.30)	44 (50.57)	
	30+	78 (16.53)	22 (13.75)	55 (11.32)	6(6.90)	
Stage	Localized	371 (78.60)	114 (71.25)	368 (75.72)	63 (72.41)	0.1623
	Metastatic	101 (21.40)	46 (28.75)	118 (24.28)	24 (27.59)	

**Table 6:** Survival estimates and Hazard Ratio from multivariate analysis for bone OS patients.

Variable	Level	5-Year OS (95% CI)	10-Year OS (95% CI)	HR (95% CI)	*p*-Value
Age	0 ≤ X ≤ 14 (ref)	64 (0.6–0.7)	57 (0.5–0.6)		<0.001
	15 ≤ X ≤ 29	60 (0.5–0.6)	56 (0.5–0.6)	1 (0.85–1.18)	
	X ≥ 30	45 (0.4–0.5)	38 (0.3–0.4)	1.59 (1.29–1.96)	
Gender	Female (ref)	61 (0.5–0.7)	57 (0.5–0.6)		0.0044
	Male	57 (0.5–0.6)	50 (0.5–0.6)	1.22 (1.06–1.41)	
Tumor site	Lower (ref)	63 (0.6–0.7)	58(0.5–0.6)		<0.001
	Upper	56 (0.5–0.6)	50(0.4–0.6)	1.13 (0.92–1.39)	
	Axial	25 (0.1–0.3)	20(0.1–0.3)	2.56 (2–3.2)	
Stage	Localized (ref)	67 (0.6–0.7)	61 (0.5–0.6)		<0.001
	Metastatic	20 (0.1–0.3)	17 (0.1–0.2)	4.44 (3.8–5.2)	
Decade	1982–1991(ref)	52 (0.4–0.6)	48 (0.4–0.5)		<0.001
	1992–2001	62 (0.5–0.7)	55 (0.5–0.6)	0.61 (0.52–0.73)	
	2002–2011	60 (0.5–0.7)	54 (0.5–0.6)	0.62 (0.50–0.73)	
	2012.2021	67 (0.6–0.7)	62 (0.5–0.7)	0.33 (0.25–0.43)	
Geographic origin	North (ref)	57 (0.5–0.6)	53 (0.5–0.6)		0.1526
	Center	58 (0.5–0.6)	51 (0.4–0.5)	1 (0.9–1.33)	
	South and islands	60 (0.5–0.6)	55 (0.5–0.6)	0.9 (0.8–1.05)	

CI (Interval of Confidence); HR (Hazard Ratio).

**Table 7:** Survival estimate by decade according to tumor stage in bone OS patients.

	1982–1991		1992–2001		2002–2011		2012–2021	
	5-Year OS (95% CI)	*p*-Value	5-Year OS (95% CI)	*p*-Value	5-Year OS (95% CI)	*p*-Value	5-Year OS (95% CI)	*p*-Value
Localized	58 (0.53–0.62)	<0.01	71 (0.67–0.75)	<0.01	70 (0.66–0.75)	<0.01	76 (0.68–0.83)	<0.01
Metastatic	10 (0.04–0.18)		21 (0.14–0.3)		15 (0.08–0.24)		40 (0.27–0.52)	

CI (Interval of confidence).

**Table 8:** Survival estimate and Hazard Ratio from multivariate analysis for EwS patients.

Variable	Level	5-Year OS (95% CI)	10-Year OS (95% CI)	HR (95% CI)	*p*-Value
Age	0 ≤ X ≤ 14	62 (0.6–0.7)	63 (0.6–0.7)		<0.001
	15 ≤ X ≤ 29	53 (0.5–0.6)	48 (0.4–0.5)	1.45 (1.2–1.8)	
	X ≥ 30	51 (0.4–0.6)	44 (0.4–0.5)	2.01 (1.6–2.6)	
Gender	Female	61 (0.6–0.7)	56 (0.5–0.6)		0.5281
	Male	59 (0.5–0.6)	53 (0.5–0.6)	1 (0.9–1.3)	
Tumor site	Axial (ref)	51 (0.5–0.6)	46 (0.4–0.5)		0.0003
	Lower	65 (0.6–0.7)	60 (0.5–0.7)	0.68 (0.6–0.8)	
	Upper	66 (0.6–0.7)	57 (0.5–0.7)	0.79 (0.6–1)	
Stage	Localized	68 (0.6–0.7)	62 (60–65)		<0.001
	Metastatic	29 (0.2–0.4)	24 (20–31)	3.78 (3.1–4.6)	
Decade	1982–1991	46 (0.4–0.5)	39 (0.3–0.5)		<0.001
	1992–2001	61 (0.6–0.7)	54 (0.5–0.6)	0.52 (0.4–0.7)	
	2002–2011	63 (0.6–0.7)	58 (0.5–0.6)	0.43 (0.3–0.5)	
	2012–2021	65 (0.6–0.7)	60 (0.5–0.6)	0.30 (0.2–0.4)	
Geographic origin	North	61 (0.6–0.7)	56 (0.5–0.6)		0.2545
	Center	60 (0.5–0.7)	54 (0.5–0.6)	0.96(0.7–1.2)	
	South and islands	56 (0.5–0.6)	50 (0.5–0.6)	1.14 (0.9–1.4)	

CI (Interval of Confidence); HR (Hazard Ratio).

**Table 9:** Survival estimate by decade according to tumor stage for EwS patients.

	1982–1991		1992–2001		2002–2011		2012–2021	
	5-Year OS (95% CI)	*p*-Value	5-Year OS (95% CI)	*p*-Value	5-Year OS (95% CI)	*p*-Value	5-Year OS (95% CI)	*p*-Value
Localized	55 (0.5–0.6)	<0.01	68 (0.6–0.7)	<0.01	73 (0.7–0.8)	<0.01	81 (0.7–0.9)	<0.01
Metastatic	3 (0.02–0.11)		31 (0.2–0.4)		33 (0.2–0.4)		33 (0.2–0.4)	

CI (Interval of confidence).

## Data Availability

The data that support the findings of this study are not publicly available due to their containing information that could compromise the privacy of research participants but are available from the corresponding author (R.L.) upon reasonable request.

## References

[1] Rojas G.A., Hubbard A.K., Diessner B.J., Ribeiro K.B., Spector L.G. (2021). International Trends in Incidence of Osteosarcoma (1988–2012). Int. J. Cancer.

[2] Klein M.J., Siegal G.P. (2006). Osteosarcoma. Am. J. Clin. Pathol..

[3] Hameed M., Dorfman H. (2011). Primary Malignant Bone Tumors-Recent Developments. Semin. Diagn. Pathol..

[4] Athanasou N., Bielack S., de Alava E., Dei Tos A.P., Ferrari S., Gelderblom H., Grimer R., Sundby Hall K., Hassan B., Hogendoorn P.C.W. (2010). Bone Sarcomas: ESMO Clinical Practice Guidelines for Diagnosis, Treatment and Follow-Up. Ann. Oncol..

[5] Strauss S.J., Frezza A.M., Abecassis N., Bajpai J., Bauer S., Biagini R., Bielack S., Blay J.Y., Bolle S., Bonvalot S. (2021). Bone Sarcomas: ESMO–EURACAN–GENTURIS–ERN PaedCan Clinical Practice Guideline for Diagnosis, Treatment and Follow-up. Ann. Oncol..

[6] Mirabello L., Troisi R.J., Savage S.A. (2009). Osteosarcoma Incidence and Survival Rates from 1973 to 2004: Data from the Surveillance, Epidemiology, and End Results Program. Cancer.

[7] Casali P.G., Blay J.Y., Bertuzzi A., Bielack S., Bjerkehagen B., Bonvalot S., Boukovinas I., Bruzzi P., Tos A.P.D., Dileo P. (2014). Bone Sarcomas: ESMO Clinical Practice Guidelines for Diagnosis, Treatment and Follow-Up. Ann. Oncol..

[8] Sbaraglia M., Bellan E., Dei Tos A.P. (2021). The 2020 WHO Classification of Soft Tissue Tumours: News and Perspectives. Pathologica.

[9] Durer S., Shaikh H. (2023). Ewing Sarcoma.

[10] Balamuth N.J., Womer R.B. (2010). Ewing‘s Sarcoma. Lancet Oncol..

[11] Coviello V., Buzzoni C., Fusco M., Barchielli A., Cuccaro F., De Angelis R., Giacomin A., Luminari S., Randi G., Mangone L. (2017). Survival of cancer patients in Italy. Epidemiol Prev..

[12] Wang X.Q., Goytain A., Dickson B.C., Nielsen T.O. (2022). Advances in Sarcoma Molecular Diagnostics. Genes Chromosom.Cancer.

[13] Grünewald T.G., Alonso M., Avnet S., Banito A., Burdach S., Cidre-Aranaz F., Di Pompo G., Distel M., Dorado-Garcia H., Garcia-Castro J. (2020). Sarcoma Treatment in the Era of Molecular Medicine. EMBO Mol. Med..

[14] Picci P., Sangiorgi L., Zavatta M., Caldora P. (1994). Register of Primary Malignant Tumors of the Bone at the Rizzoli Orthopaedic Institute in Bologna. Chir. Organi Mov..

[15] Siegal G.P.H.M., Bloem J.L., Cates J.M.M. (2013). WHO Classification of Tumours of Soft Tissue and Bone.

[16] Di Zio M., Ferruzza A. (2024). L’informazione Statistica Microterritoriale: L’esperienza del Registro Statistico di Base dei Luoghi.

[17] ISTAT (2022). Territorio 2022.

[18] ISTAT (2020). Descrizione Dei Dati Geografici Dei Confini Delle Unità Amministrative a Fini Statistici.

[19] Raedkjaer M., Maretty-Kongstad K., Baad-Hansen T., Safwat A., Mørk Petersen M., Keller J., Vedsted P. (2020). The Association between Socioeconomic Position and Tumour Size, Grade, Stage, and Mortality in Danish Sarcoma Patients—A National, Observational Study from 2000 to 2013. Acta Oncol..

[20] Alsoof D., Kasthuri V., Homer A., Glueck J., McDonald C.L., Kuris E.O., Daniels A.H. (2023). County Rurality Is Associated with Increased Tumor Size and Decreased Survival in Patients with Ewing Sarcoma. Orthop. Rev..

[21] Wendt R., Gao Y., Miller B.J. (2019). Rural Patients Are at Risk for Increased Stage at Presentation and Diminished Overall Survival in Osteosarcoma. Cancer Epidemiol..

[22] Lozano-Calderón S.A., Albergo J.I., Groot O.Q., Merchan N.A., El Abiad J.M., Salinas V., Gomez Mier L.C., Montoya C.S., Ferrone M.L., Ready J.E. (2023). Complete Tumor Necrosis after Neoadjuvant Chemotherapy Defines Good Responders in Patients with Ewing Sarcoma. Cancer.

[23] Ritter J., Bielack S.S. (2010). Osteosarcoma. Ann. Oncol..

[24] Wilson R., Reinke D., van Oortmerssen G., Gonzato O., Ott G., Raut C.P., Guadagnolo B.A., Haas R.L.M., Trent J., Jones R. (2024). What Is a Sarcoma ‘Specialist Center’? Multidisciplinary Research Finds an Answer. Cancers.

[25] Ottesen T.D., Shultz B.N., Munger A.M., Sibindi C., Yurter A., Varthi A.G., Grauer J.N. (2022). Characteristics, Management, and Outcomes of Patients with Osteosarcoma: An Analysis of Outcomes from the National Cancer Database. J. Am. Acad. Orthop. Surg. Glob. Res. Rev..

[26] Verma V., Denniston K.A., Lin C.J., Lin C. (2017). A Comparison of Pediatric vs. Adult Patients with the Ewing Sarcoma Family of Tumors. Front. Oncol..

[27] Cole S., Gianferante D.M., Zhu B., Mirabello L. (2022). Osteosarcoma: A Surveillance, Epidemiology, and End Results Program-Based Analysis from 1975 to 2017. Cancer.

[28] Lee J., Hoang B.H., Ziogas A., Zell J.A. (2010). Analysis of Prognostic Factors in Ewing Sarcoma Using a Population-Based Cancer Registry. Cancer.

[29] Mirabello L., Troisi R.J., Savage S.A. (2009). International Osteosarcoma Incidence Patterns in Children and Adolescents, Middle Ages and Elderly Persons. Int. J. Cancer.

[30] Bosma S.E., Ayu O., Fiocco M., Gelderblom H., Dijkstra P.D.S. (2018). Prognostic Factors for Survival in Ewing Sarcoma: A Systematic Review. Surg. Oncol..

[31] Benigni R. (2007). Social Sexual Inequality and Sex Difference in Cancer Incidence. Environ. Res..

[32] Picci P., Osteogenic O. (2007). Osteosarcoma (Osteogenic Sarcoma) Disease Name and Synonyms Definition and Diagnostic Criteria. Orphanet J. Rare Dis..

[33] Narayanan G., Kamala L.H., Nair S.G., Purushothaman P.N., Kumar A., Kattoor J. (2024). Ewing’s Sarcoma in Adolescents and Adults-10-Year Experience from a Tertiary Cancer Center in India. J. Cancer Res. Ther..

[34] Ruhstaller T., Roe H., Thürlimann B., Nicoll J.J. (2006). The Multidisciplinary Meeting: An Indispensable Aid to Communication between Different Specialities. Eur. J. Cancer.

[35] Kaste S.C. (2011). Imaging Pediatric Bone Sarcomas. Radiol. Clin. N. Am..

[36] Anninga J.K., Gelderblom H., Fiocco M., Kroep J.R., Taminiau A.H.M., Hogendoorn P.C.W., Egeler R.M. (2011). Chemotherapeutic Adjuvant Treatment for Osteosarcoma: Where Do We Stand?. Eur. J. Cancer.

[37] Brennan B., Kirton L., Marec-Bérard P., Gaspar N., Laurence V., Martín-Broto J., Sastre A., Gelderblom H., Owens C., Fenwick N. (2022). Comparison of Two Chemotherapy Regimens in Patients with Newly Diagnosed Ewing Sarcoma (EE2012): An Open-Label, Randomised, Phase 3 Trial. Lancet.

[38] Gherlinzoni F., Picci P., Bacci G., Campanacci D. (1992). Limb Sparing versus Amputation in Osteosarcoma: Correlation between Local Control, Surgical Margins and Tumor Necrosis: Istituto Rizzoli Experience. Ann. Oncol..

[39] Papakonstantinou E., Stamatopoulos A., I Athanasiadis D., Kenanidis E., Potoupnis M., Haidich A.-B., Tsiridis E. (2020). Limb-Salvage Surgery Offers Better Five-Year Survival Rate than Amputation in Patients with Limb Osteosarcoma Treated with Neoadjuvant Chemotherapy. A Systematic Review and Meta-Analysis. J. Bone Oncol..

[40] Ottaviani G., Jaffe N. (2009). The Epidemiology of Osteosarcoma. Cancer Treat. Res..

[41] Duchman K.R., Gao Y., Miller B.J. (2015). Prognostic Factors for Survival in Patients with Ewing’s Sarcoma Using the Surveillance, Epidemiology, and End Results (SEER) Program Database. Cancer Epidemiol..

